# The Impact of O-Glycosylation on Cyanidin Interaction with POPC Membranes: Structure-Activity Relationship

**DOI:** 10.3390/molecules23112771

**Published:** 2018-10-25

**Authors:** Sylwia Cyboran-Mikołajczyk, Piotr Jurkiewicz, Martin Hof, Halina Kleszczyńska

**Affiliations:** 1 Department of Physics and Biophysics, Wroclaw University of Environmental and Life Sciences, Norwida 25, 50-375 Wrocław, Poland; halina.kleszczynska@upwr.edu.pl; 2 J. Heyrovsky Institute of Physical Chemistry, Academy of Science of the Czech Republic, Dolejškova 2155/3, 8, 182 23 Prague, Czech Republic; piotr.jurkiewicz@jh-inst.cas.cz (P.J.); hof@jh-inst.cas.cz (M.H.)

**Keywords:** structure-activity relationships, lipid peroxidation, phospholipid membrane, anthocyanin, fluorescence dyes, hydrogen peroxide, membrane fluidity

## Abstract

Cyanidin and its O-glycosides have many important physiological functions in plants and beneficial effects on human health. Their biological activity is not entirely clear and depends on the structure of the molecule, in particular, on the number and type of sugar substituents. Therefore, in this study the detailed structure-activity relationship (SARs) of the anthocyanins/anthocyanidins in relation to their interactions with lipid bilayer was determined. On the basis of their antioxidant activity and the changes induced by them in size and Zeta potential of lipid vesicles, and mobility and order of lipid acyl chains, the impact of the number and type of sugar substituents on the biological activity of the compounds was evaluated. The obtained results have shown, that 3-O-glycosylation changes the interaction of cyanidin with lipid bilayer entirely. The 3-O-glycosides containing a monosaccharide induces greater changes in physical properties of the lipid membrane than those containing disaccharides. The presence of additional sugar significantly reduces glycoside interaction with model lipid membrane. Furthermore, O-glycosylation alters the ability of cyanidin to scavenge free radicals. This alteration depends on the type of free radicals and the sensitivity of the method used for their determination.

## 1. Introduction

Cyanidin as an aglycone, i.e., without sugar substituents, belongs to the anthocyanins and occurs in Nature very rarely. On the other hand its glycosides—anthocyanidins—are the most common plant colorants. Their presence in fruits and vegetables makes them abundant in the human diet. The monosaccharide and disaccharide moieties are usually connected with cyanidin through O-glycosidic bonds at C_3_-OH or at C_3_-OH and C_5_-OHs [1,2,3]. The most common glycosyl units of cyanidin are monosaccharides: glucose, galactose, rhamnose, xylose and arabinose, and homogenous or heterogeneous disaccharides: rutinose, sophorose, sambudiose. Glucose is the most common among all the abovementioned glycosyl units, and is strongly associated with the stability, absorption and bioavailability of anthocyanins [4]. The number, type and position of a sugar in their structure can significantly change their physicochemical properties as well as their biological activity. From the physical point of view glycosylation of cyanidin affects the polarity, size and structure of the molecule. From the chemical point of view glycosylation can reduce the partition of the compound into non-polar media and also, due to increased polarity, alter the access to lipid phase and hydrophobic free radicals [5]. Additionally, by reducing the number of free OH groups, glycosylation can decrease the ability of molecules to delocalize electrons and decrease their metal chelating properties [6,7]. On the other hand, glycosylation increases pH- and temperature-stability and consequently protects the molecule from degradation under different conditions [8,9]. Since glycosylation results in the formation of the intramolecular H-bond networks, it decreases the hydrolysis rate [9,10], and changes the activation energy of fading. Therefore, it protects the color forms of anthocyanidins from degradation [11].

Both in vivo and in vitro investigations have shown that cyanidin and its glycosides have many crucial physiological functions in plants, as well as pharmaceutical activities in humans. They are responsible for the color of plant tissues and their protection against many abiotic stressors, prolonging the lifetime of the plants in adverse conditions [12]. In relation to human health, they exhibit not only protective but also therapeutic effects due to their activities i.e., antioxidant, anti-inflammatory and anticancer [13,14]. As mentioned above, the physicochemical properties of cyanidin and its glycosides depend on the number, type and position of sugars in their structures, which determine their biological activity. Literature data indicate that their bioavailability could also significantly depend on the sugar moiety, which determines their recovery within the gastrointestinal tract [15]. Despite this, the structure-activity relationship (SARs) of the anthocyanins/anthocyanidins is not entirely clear, and contradictory findings are often published. A good example is their antioxidant activity that was found to be increased or decreased as a result of the glycosylation [16]. In order to better understand the impact of the O-glycosylation on the biological activity of anthocyanidins, the basic biophysical studies should be carried out. Therefore, the studies of the influence of cyanidin and five of its derivatives on the physical properties of 1-palmitoyl-2-oleoylphosphatidylcholine (POPC) lipid membrane were undertaken in order to establish the SARs. POPC is commonly used in membrane studies as it mimics mammalian phospholipid composition well [17,18]. To the best of our knowledge, in the literature there are only a few studies concerning the interaction of cyanidin and its glycosides with lipid membranes. Rokic et al. investigated the interaction of cyanidin and cyanidin 3-O-β-glucospyranoside with DPPC and DPPG membrane [19]. The impact of cyanidin, cyanidin-3-O-glucoside, cyanidin-3-5-di-O-β-glucoside, and cyanidin-3-O-β-(6′-O-*E*-*p*-cumaroylsambubioside)-5-O-β-glucoside on cancer-mimic membrane was also studied [20]. There is a lack of studies comparing the activity of cyanidin and its glycosides, containing various sugar substituents, that would determine the relationship between the structure and activity of these compounds in relation to lipid bilayer. This type of basic research is necessary to determine the molecular mechanism responsible for the biological activity of the compounds, because the lipid-protein membrane constitute the first barrier, which determines the interactions of different substances with organisms. Understanding the effects of these compounds on the physical properties of the membrane allows to explain the mechanism responsible for their well-documented biological activity, at the molecular and cellular level. Moreover, knowing the mechanism of their interaction with lipid membranes allows a better understanding of their transport across these membranes, and thus, their bioavailability.

Our studies were performed for cyanidin (C) and five of its O-glycosides containing one or two monosacharides (CG—cyanidin-3-O-glucoside, CGA—cyanidin-3-O-galactoside, CA—cyanidin-3-O-arbinoside, CDG—cyanidin-3-5-O-di-glucoside) and disaccharide (CR—cyanidin-3-O-rutinoside). In this work the antioxidant activity of the compounds was determined by studying 2,2′-azobis(2-amidinopropane) dihydrohloride (AAPH) andH_2_O_2_ induced oxidation of POPC membrane using fluorimetric methods. Furthermore, the impact of the compounds on the physical properties of the membrane was determined by using steady-state and time-resolved fluorimetry, and dynamic and electrophoretic light scattering methods. The influence of the compounds on the arrangement of the polar heads of lipids, and the mobility and order of lipid acyl chains were determined. Furthermore, we examined the ability of the compounds to induce aggregation of liposomes and changes in membrane Zeta potential. On the basis of the obtained results the impact of the type and number of sugar substituent on biological activity of cyanidin was determined.

## 2. Results and Discussion

### 2.1. The Impact of the Compounds on the Physical Properties of Lipid Membrane—Fluorescence Spectroscopy Studies

The fluorescent probes, Laurdan and diphenylhexatriene (DPH), were used to characterize the interaction of the studied compounds with POPC liposomes. The fluorophore of the Laurdan probe is connected to the lauryl chain and is located at the level of the phospholipid glycerol backbone [21,22]. The DPH probe is located in the area occupied by the hydrocarbon chains of lipids. Its long axis is approximately aligned along the bilayer normal [23]. In the first step, it was checked whether the addition of any of the studied compounds would result in fluorescence quenching of the probes. For this purpose the fluorescence intensities of the probes at 440 nm were measured before (*I_0_*) and after (*I*) the addition of the tested compounds at room temperature. The Stern-Volmer plot obtained for Laurdan probe (Supplementary Material, Figure S1) describing the relationship between the ratio of the fluorescence intensities (*I*_0_/*I*) and the concentration of the compounds clearly indicates that cyanidin glycosides at concentrations higher than 25 µM quench Laurdan fluorescence. Decrease in fluorescence intensity of Laurdan probe caused by aglycone (C) is much stronger than for its glycosides (Supplementary Material, Figure S1). On the basis of the obtained results the ability of the compounds to quench Laurdan fluorescence can be summarized as follow: CDG < CA = CGA ≤ CG ≤ CR < C. There was no correlation between the Laurdan quenching capacity and the total number of hydroxyl groups in sugar or per molecule. Therefore, these results indicate that the O-glycosylation of cyanidin significantly inhibits its ability to static and/or dynamic quenching of Laurdan fluorescence probably by limiting the penetration of this compound into the membrane interior. Because of the compounds-induced strong quenching of Lauran probe, the use of the probe to examine their impact on the physical properties of the hydrophilic part of the membrane was excluded.

**Figure 1:**
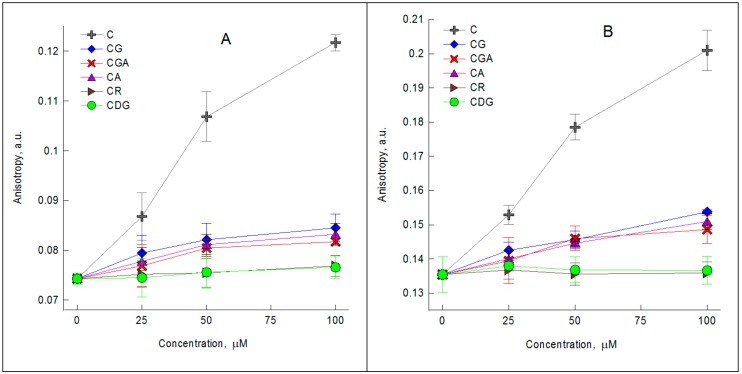
The values of anisotropy of diphenylhexatriene (DPH) probe determined for 1-palmitoyl-2-oleoylphosphatidylcholine (POPC) large unilamellar vesicles without and with addition of 25 µM, 50 µM and 100 µM of cyanidin and its glycosides at: (**A**) 35 °C and (**B**) 15 °C.

In the case of DPH probe, the tested compounds do not influence the fluorescence intensity emitted at 425 nm (i.e., no quenching was detected), and therefore this probe was used in the studies on the effects of the tested compounds on the physical properties of the hydrophobic part of the lipid membrane. DPH anisotropy allows estimation of the lipid chain order and mobility, often referred to as fluidity of the lipid bilayer [24]. The steady-state measurements with DPH probe have shown that the used compounds may affect the fluidity of the hydrophobic part of the membrane, especially when used at concertation higher than 25 µM. The changes in the fluorescence anisotropy of the DPH probe induced by increasing concentration of the tested compounds at 35 °C and 15 °C are shown in Figure 1A,B. CDG and CR did not cause any changes, whilst for aglycone (C) and glycosides containing monosaccharides (CA, CGA, CG) there was a concentration-dependent increase in anisotropy of DPH. This increase of anisotropy suggests that these compounds reduce fluidity of the area of the hydrocarbon chains of membrane lipids. Taking into account the changes induced by the compounds, their ability to modify the membrane fluidity may be described as follows: CDG = CR < CGA = CA = CG < C. The results clearly indicate that: (1) C causes the greatest changes in hydrophobic part of the membrane; (2) the 3-O-glycosylation of cyanidin significantly limits its ability to modify the hydrophobic membrane region, whilst the presence of an additional sugar group at C_5_ abolishes it completely; (3) cyanidin glycosides containing a monosaccharide (CGA, CA, CG) induces greater changes in the hydrophobic part of the membrane than those containing a disaccharide (CR). Those observations have been confirmed by the Pearson correlation analysis (Supplementary Materials, Figure S2). It has shown a significant negative correlation between the anisotropy increase and the total number of OH groups in the structure of the compounds.

**Figure 2:**
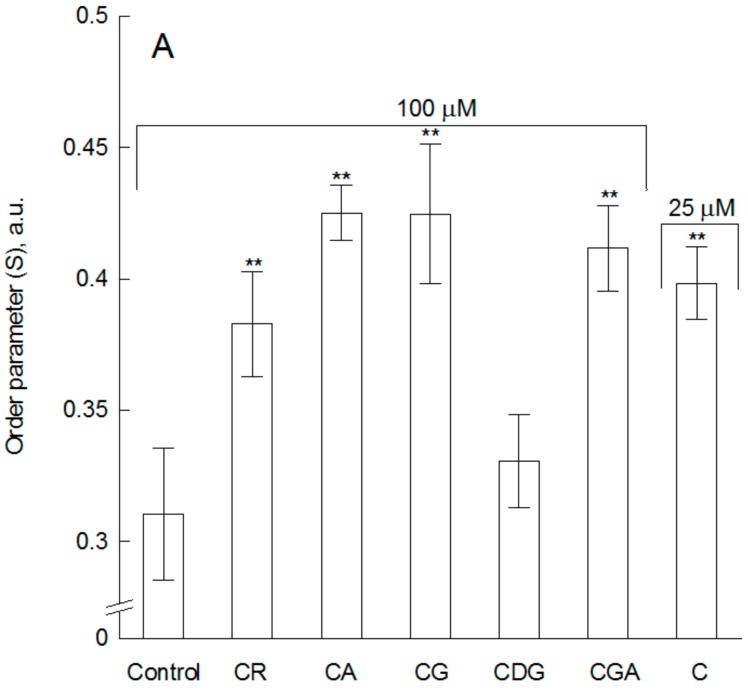

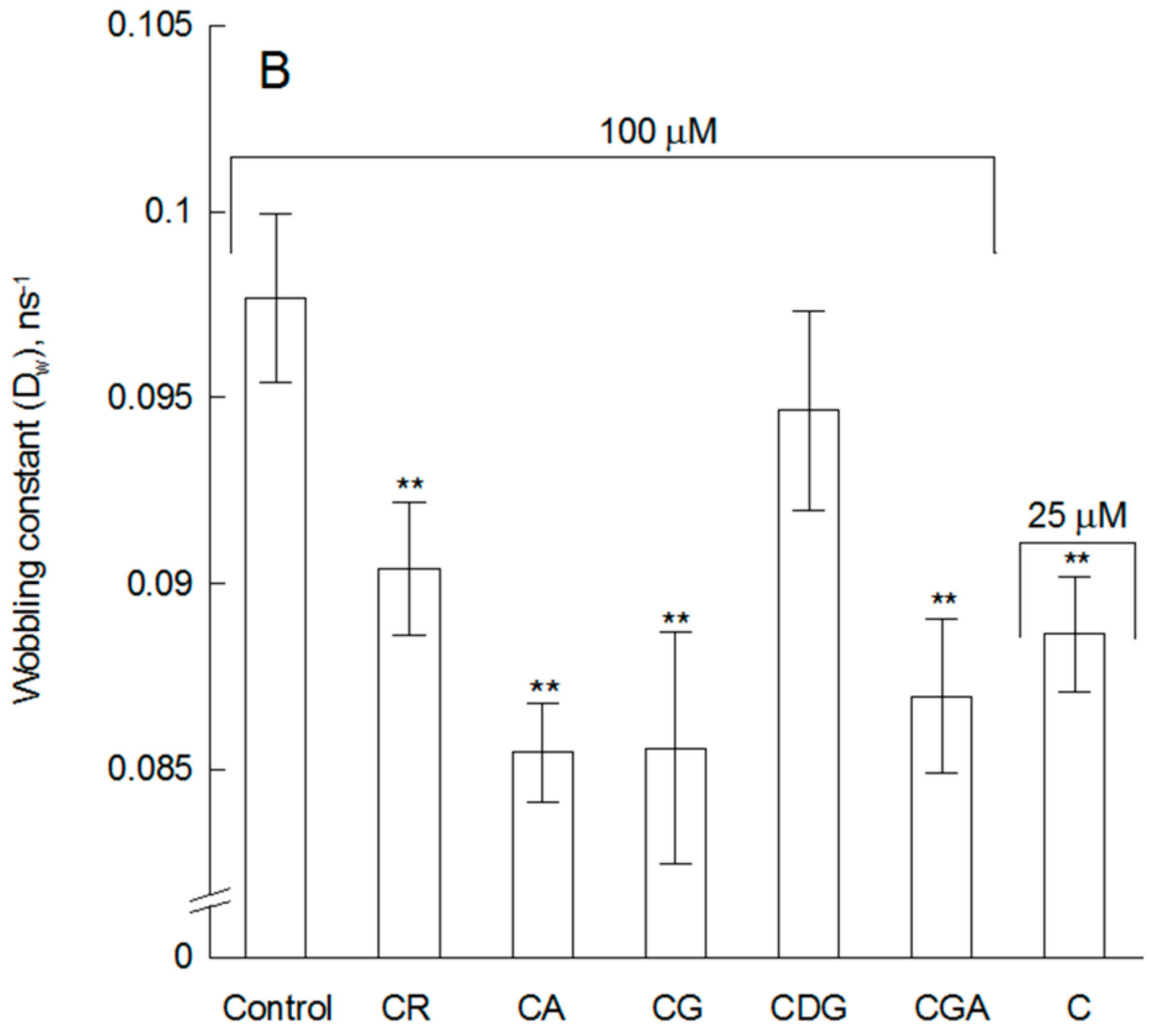
Order parameter (S) (**A**) and wobbling constant (*D*_w_) (**B**) of DPH probe determined for POPC membrane and POPC with addition of cyanidin used at 25 µM and its glycosides used and 100 µM. Statistically significant differences between values determined for control and modified membrane were marked: ** at α = 0.01.

Based on time-resolved anisotropy decays of DPH probe, *S* and *D_w_* parameters were calculated for POPC large unilamellar vesicels (LUVs) in the absence and in the presence of the tested compounds at 15 °C (Equations (4) and (5)). This method allows us to distinguish between structural and dynamic effects. The order parameter of DPH probe (*S*) approximates the lipid acyl chain order [25], while DPH wobbling diffusion coefficient (*D_w_*) describes their dynamics. The increase of the order parameter and the decrease of wobbling constant was observed under the influence of the tested compounds (Figure 1). Similar to the results obtained in steady-state measurements, at concentration of 25 µM, cyanidin glycosides practically do not induce any changes in *S* and *D_w_* values. At a higher concentration of 100 µM the changes are statistically significant, with exception of CDG, which is completely inactive (Figure 2A,B). Additionally, the changes in *S* and *D_w_* induced by cyanidin (C) used at a concentration of 25 µM are similar to those induced by its monoglycosides (CA, CG, CGA, CR) used at four time higher concentration (100 µM).

The strength of the influence of the studied compounds on the ordering and mobility of the hydrophobic core of the lipid bilayer is as follow: CDG < CR < CGA = CA = CG < C. These results are in good agreement with the results obtained by steady state methods. Furthermore, they additionally explain that the observed decrease in membrane fluidity in the presence of the compounds is a result of both increased ordering and limited mobility of the acyl chains of lipids.

Our results are in a good agreement with literature data that showed a decrease of membrane fluidity under the influence of cyanidin and other flavonoids [20,26] and that the glycosylation may significantly reduce the membrane interactivity of flavonoids [26,27]. Cyanidin quenches Laurdan fluorescence much stronger than its glycosides, indicatin its higher affinity for the lipid bilayer. However, it should be also taken into account that, all of the used compounds may partially absorb the fluorescence emitted by this probe, because their absorbance maxima in the solution at neutral pH, are between 550–600 nm [28,29]. Cyanidin causes considerable changes in the deeper regions of the lipid bilayers, so it is likely localized in the hydrophobic-hydrophilic interphase near the chromophore of Laurdan probe, hence both static and collisional quenching of the probe may occur. Furthermore, changes in DPH fluorescence indicate that it is highly likely that cyanidin may also partially incorporate to the hydrophobic core of the POPC membrane. In the case of glycosides, the quenching of Laurdan fluorescence and only slight stiffening of the hydrocarbon chains of lipids indicate that O-glycosides containing monosaccharide (CA, CG, CGA) may interact mainly with the polar heads of lipids. The head-group preference of these compounds is likely caused by the presence of glycosyl -OH groups in their structure, which can easily form H-bonds with the surrounding water molecules and thereby increase compounds hydrophilicity [5]. Additionally, it was demonstrated also for other flavonoids, that the size of the sugar moiety can significantly restrict their partition into water-lipid interface and in turn limit glycosidies penetration into the deeper membrane regions [26,30]. Their location in hydrophilic membrane area, in consequence, leads to very little alteration observed in the hydrophobic core of the membrane. Furthermore, the substitution of cyanidin with disaccharide (CR) restricts its influence on the hydrophobic part of the membrane to a greater extent than with monosaccharide, probably due to the different special structure and the greater size of the molecule. The presence of additional sugar group at C_5_ position (CDG) completely limits interaction of cyanidin O-glycosides with the lipid membrane. It means that 3-O-glycosides containing disaccharides (CR) and 3-5-O-di-glycosides (CDG) bind also to the polar part of the membrane or remain in the water phase adjacent to the membrane surface.

### 2.2. The Impact of the Compounds on the Physical Properties of Lipid Membrane—Dynamic and Electrophoretic Light Scattering Studies

**Figure 3:**
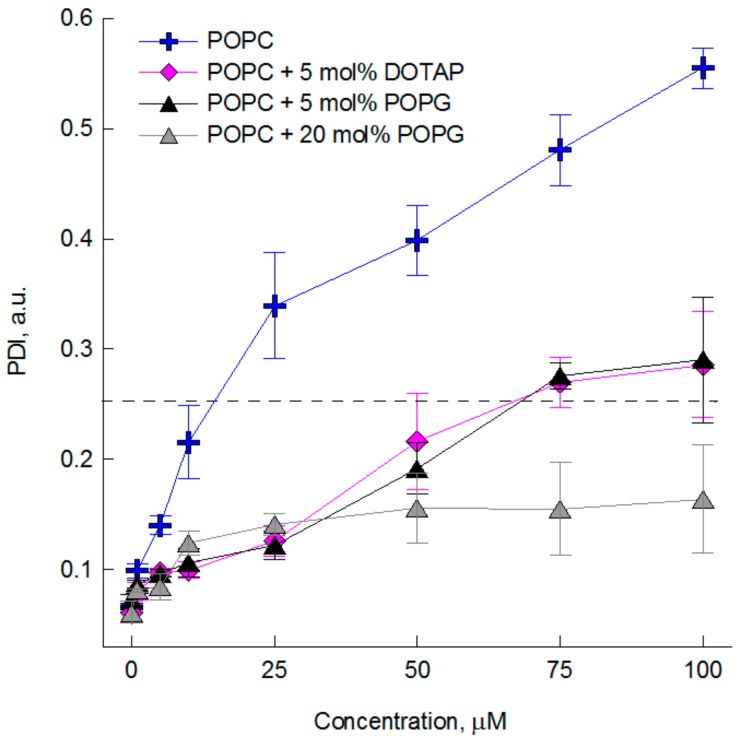
The polydispersity index (PDI) values of LUVs formed from zwitteronic lipids (POPC) and with positive (POPC-DOTAP) and negative (POPC-POPG) charge net. The measurements were performed 15 min after cyanidin addition (10–100 µM). Results are shown as mean ± SD. PDI above which the liposome aggregation was observed is marked with a dashed line. POPG is 1-palmitoyl-2-oleoyl-sn-glycero-3-phospho-glycerol and DOTAB is 1,2-dioleoyl-3-trimethylammonium-propane.

To investigate the influence of the studied compounds on the size of large unilamellar POPC vesicles dynamic light scattering (DLS) measurements were performed. On the basis of intensity-size distribution profiles of POPC vesicles unmodified and modified by the compounds the mean size (Z-AVG) of lipid vesicles and polydispersity index (PDI) were determined. The obtained results have shown that none of the studied cyanidin glycosides affect the size of lipid vesicles. The average diameter and PDI, determined for these compounds used at concentration of 50 µM (10 mol %), did not differ from those determined for non-modified liposomes and do not change over time (Z-AVG = 137 ± 7.2 nm, PDI = 0.068 ± 0.015). Aglycone (C), used in the same concentration, caused rapid aggregation of liposomes, that was observed within 15 minutes after its addition (Z-average = 212 ± 57, PDI = 0.389 ± 0.050). In this case, the three fractions of liposome volume-weighted sizes were observed with a diameter of 160 nm, 730 nm and 5450 nm, that constitute about 25, 15, and 60% of volume, respectively. In order to determine the maximum concentration of cyanidin that does not cause aggregation of POPC liposomes Z-average and PDI were determined 15 and 120 min after its addition (Figure 3, Table S1). Figure 3 shows a relationship between PDI and the concentration of cyanidin measured 15 min after its addition. Below 10 µM (after 15 min) and 1 µM (after 120 min) we found no signs of liposome aggregation, i.e., the system was monodisperse with PDI <0.1 and mean diameters very similar to that of unmodified liposomes. At 10 µM (after 15 min) and 1 µM (after 120 min) an increase of both PDI and Z-Average was observed, but the suspension was still relatively homogeneous (PDI < 0.25). For higher concentrations PDI have increased significantly.

Literature data indicate, that some polyphenols can cause the aggregation of liposomes [31,32], but the mechanism responsible for it may be different. Lehtonen et al. [31] showed that the isoflavone daidzein causes strong aggregation of liposomes composed of phospholipids with a net negative charge. This behavior was attributed to membrane dehydration by daidzein, which was anchored at the border between hydrophobic and hydrophilic area of the membrane [31]. On the other hand, the mechanism of polyphenol-induced aggregation of liposomes may be based on the existence of polyphenolic bridges between adjacent surfaces. These bridges decrease repulsive hydration forces between bilayers and attract bilayer surfaces thanks to hydrogen bonding [32,33]. Therefore, in order to better understand the mechanism responsible for the cyanidin-induced liposomes aggregation the DLS measurements were also conducted for POPC liposomes -containing 1-palmitoyl-2-oleoyl-sn-glycero-3-phospho-glycerol (POPC + 5 or 20 mol % POPG) and 1,2-dioleoyl-3-trimethylammonium-propane -(POPC + 5 mol % DOTAP). The results have shown that C may cause the aggregation of liposomes of both slightly positive (POPC-DOTAP 5 mol %) and slightly negative (POPC-POPG 5 mol%) net charge, but only at concentrations much higher than those causing aggregation of pure POPC membrane (Figure 3). The fourfold increase of the negative net charge of the lipid membrane (POPC-POPG 20 mol %) completely inhibits the cyanidin-induced aggregation process of the liposomes. The obtained relationship between the net charge of the membranes and their aggregation caused by C shows that the interaction of this compounds with lipid membrane strongly depends on lipid composition. Cyanidin at pH 7.4 occurs in a natural four forms: quinone base, hemicetal, pseudobase and chalcone [34], therefore, the demonstrated results indicate its weaker interaction with charged groups of lipids, in accordance with results published in [19].

**Figure 4:**
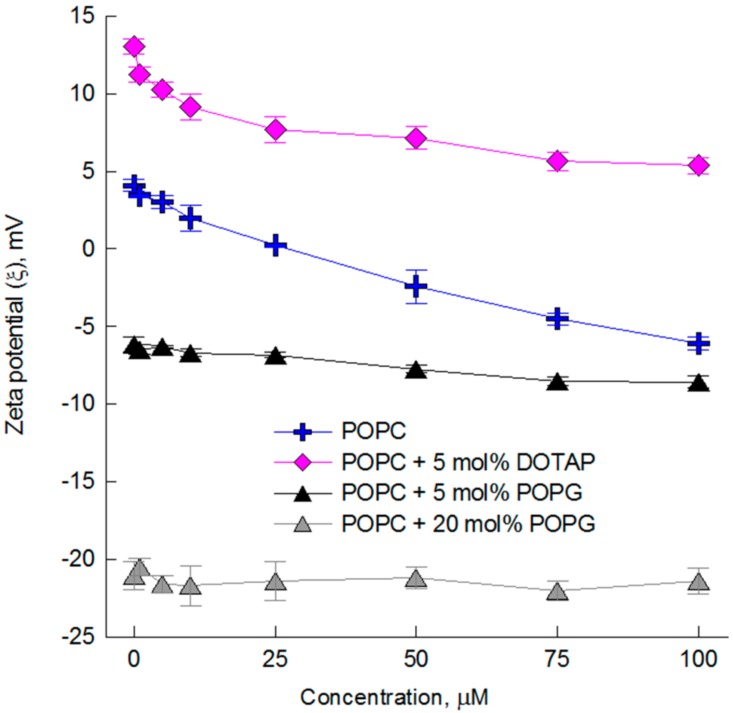
The Zeta potential values of LUVs liposomes formed from zwitteronic lipids (POPC) and with positive (POPC-DOTAP) and negative (POPC-POPG) charge net. The measurements were done 15 min after modification of liposomes by cyanidin used at different concentrations (10–100 µM). The experiment was repeated thrice and the results are shown as mean value ± standard deviation.

In order to confirm the weaker interaction of C with the charged lipids, the impact of cyanidin on Zeta potential of liposomes was determined by using electrophoretic light scattering methods (ELS). This research was also conducted for cyanidin glycosides but only in relation to the membrane composed of zwitterionic POPC lipids. The results have shown, as supposed, that cyanidin glycosides do not induce changes in the Zeta potential of POPC membrane (Figure S3, Supplementary Materials). The initial slight positive Zeta potential (+4.5 mV) of POPC liposomes that can be seen in Figure 4, results probably from the influence of ionic strength of phosphate buffer and temperature on the orientation of the polar heads of lipids [35,36].

Cyanidin presence decreases the Zeta potential of POPC and POPC-DOTAP membranes when used at concentrations higher than 25 µM (Figure 4). Furthermore, observed changes are slightly higher for zwitterionic membrane (POPC) that for positively charged one (POPC-DOTAP). In the case of membranes with negative net charge, the only slight decrease in Zeta potential was observed for POPC-POPG (5 mol %) containing membranes and the lack of changes in Zeta potential of membranes of higher negative charge (20 mol % of POPG). The former results are in a good agreement with DLS studies, because the low value of zeta potential (about −20 mV), should stabilize the liposomes and protect them against aggregation [37]. These results indicate and confirm that interaction of cyanidin with charged lipids is limited. In POPC-PGPC membranes, POPG lipids were found to be involved in hydrogen bonding with POPC lipids. As a consequence, the hydration of the mixed bilayer is different from that of the pure POPC, and the ordering of water molecules at longer distances was observed [38]. Therefore, the changes in hydrophilic membrane area are responsible for limited penetration of cyanidin to the hydrophilic-hydrophobic interphase and dipper hydrophobic core of the membrane. In order to determine the exact mechanism of this limited interaction additional studies are necessary.

### 2.3. The Antioxidant Activity of the Compounds—Fluorescence Spectroscopy Studies

**Figure 5:**
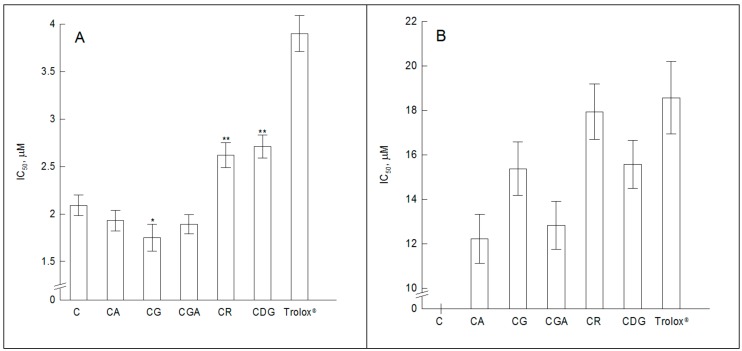
The concentrations (IC_50_) of cyanidin, its glycosides and Trolox^®^ responsible for 50% inhibition of lipid peroxidation in POPC LUVs. The oxidation of lipids was induced: (**A**) for 30 min by AAPH and (**B**) for 3 h by H_2_O_2_. In BODIPY method the determination of IC_50_ concentration of C was impossible due to its strong aggregation of POPC liposomes that occurs at effective concentration range. The statistically significant differences between antioxidant activity of C and its glycosides are denoted: * α = 0.1 and ** α = 0.05.

In this study the effect of cyanidin and its glycosides on AAPH- and H_2_O_2_-induced lipid peroxidation of POPC lipid bilayer was investigated by measuring fluorescence intensity of 1-(4-trimethylammoniumphenyl)-6-phenyl-1,3,5-hexatriene p-toluene-sulfonate (TMA-655/676 probes, respectively. The concentrations (IC_50_) responsible for 50% inhibition of lipid peroxidation were taken as a measure of antioxidant activity of the compounds. We found that all compounds effectively protect lipids against AAPH-induced oxidation and that their protection is significantly higher than these of Trolox^®^, commonly used as a standard hydrophilic antioxidant (Figure 5A). We also found that the antioxidant activity of C does not differ significantly from those of its 3-O-glycosides containing monosaccharides (CA, CG, CGA). It means that the responsible for their ability to scavenge AAPH-induced free radicals are mainly the two OH groups of B ring (at positions 3’ and 4’) and one of C ring (at C_7_). Interestingly, the results have shown that the presence of disaccharide (CR) or additional sugar substituent at C_5_ position (CDG) significantly reduces antioxidant activity of cyanidin and its glycosides containing monosaccharides. The Pearson correlation analysis has shown a significant correlation between the IC_50_ concentrations of glycosides and the total number of OH groups in the structure (Supplementary Materials, Figure S3). The presence of disaccharides or two sugar molecules change the special structure of the compound, weaker its interaction with the membrane and therefore may restrict the availability of the OH groups responsible for the scavenging of free radicals inducted by AAPH.

In the second method, lipid peroxidation was induced by H_2_O_2_. The obtained results showed that antioxidant activities of the tested compounds did not differ significantly from the antioxidant activity specified for Trolox^®^ (Figure 5B). We did not observe any significant differences between the antioxidant activity of all the used cyanidin glycosides. These results indicate that the ability of cyanidin O-glycosides to scavenge the peroxyl radicals generated by H_2_O_2_ does not depend on the type and number of sugar substituent. These results indicate, that these are the OH groups that are presented in all of the used compounds (i.e., two at positions 3’ and 4’ of B ring, and one at C_7_ of C ring) that are responsible for the removal of peroxyl radicals. Taking into account the results obtained in both used methods we can conclude that antioxidant activity of the tested compounds depends on the type of free radicals, the place of their generation (their availability for the tested compounds) and the sensitivity of the method used for their determination. Both the obtained correlation between the number of OH groups and antioxidant activity in the first method and the lack of any relationship in the second are in a good agreement with literature data [16].

## 3. Materials and Methods

### 3.1. Anthocyanin and Anthocyanidins

Cyanidin (C), cyanidin-3-O-glucoside (CG), cyanidin-3-O-galactoside (CGA), cyanidin-3-O-arabinoside (CA), cyanidin-3-O-rutinoside (CR) and cyanidin-3-5-diglucoside were purchased from Extrasynthese (Genay, France). The trade names of the compounds, chemical structure and type, place and number of sugar substituents of cyanidin are given in Table 1.

### 3.2. Lipids, Liposomes and Reagents

1-Palmitoyl-2-oleoylphosphatidylcholine (POPC), 1-palmitoyl-2-oleoyl-*sn*-glycero-3-phospho-glycerol (POPG), 1,2-dioleoyl-3-trimethylammonium-propane (DOTAB) were purchased from Avanti^®^ Polar Lipids, Inc. (Alabaster, AL, USA) Fluorescence probes diphenylhexatriene (DPH), 1-(4-trimethylammoniumphenyl)-6-phenyl-1,3,5-hexatriene *p*-toluene-sulfonate (TMA-DPH), 6-dodecanoyl-2-dimethylaminonaphthalene (Laurdan), and lipid oxidation inducers 2,2′-azobis(2-amidinopropane) dihydrohloride (AAPH) and hydrogen peroxide (H_2_O_2_) were purchased from Sigma Aldrich (Steinheim, Germany). The lipid peroxidation sensor BODIPY^TM^ 655/676 was purchased from Thermo Fisher Scientific Inc. (Waltham, MA, USA). All reagents used were of analytical grade.

### 3.3. LUVs

Large unilamellar vesicles (LUVs) were composed of POPC for all fluorimetric studies and of POPC, POPC + 5 mol % of POPG and POPC + 5 mol % of DOTAP for dynamic and electrophoretic light scattering measurements (DLS and ELS). The lipids (0.5 mM) or a mixture of lipids with fluorescence probe (100:1 molar ratio) were dissolved in chloroform. The test compounds were dissolved in ethanol and added to the lipid solutions in a molar ratios from 20:1 to 5:1 for membrane interaction studies. Next, organic solvents were thoroughly evaporated to dryness under nitrogen. Subsequently, a phosphate solution of pH 7.4 was added and multilamellar vesicles (MLVs) were formed by vortexing. The MLVs were then extruded through filters with 100 nm pores (Whatman^®^, GE Healthcare, Chicago, IL, USA), which allowed obtaining vesicles with a mean diameter of 110–120 nm. For antioxidant activity, DSL and ESL measurements the test compounds were added to the suspension of the liposomes just before measurements and incubated at least for 15 min in the dark. 

### 3.4. Cyanidin-Membrane Interaction Studies

#### 3.4.1. Steady-State Fluorescence Spectroscopy

The steady-state (SS) fluorescence was measured according to the procedure described in Cyboran et al. [39] with minor modifications. The steady-state emission spectrum (*λ*_EX_ = 373 nm) and two excitation spectra (*λ*_EM_ = 440 and 490 nm) of Laurdan probe and the emission spectra (*λ*_EX_ = 360 nm, λ_EM_ = 425 nm) of DPH probe were collected using a Fluorolog-3 spectrofluorimeter (model FL3-11, JobinYvon Inc., Edison, NJ, USA). Emission spectra of Laurdan were used to calculate the so-called generalized polarization (GP) [40] according to:

(1) $$GP=\frac{\left(I_{b}-I_{r}\right)}{\left(I_{b}+I_{r}\right)}$$

where *I*_b_ and *I*_r_ are fluorescence intensities emitted at 440 and 490 nm, respectively.

Fluorescence anisotropy (*r*_ss_) of DPH probe was calculated using the following formula [41]:

(2) $$r_{ss}=\frac{\left(I_{II}-GI_{⊥}\right)}{\left(I_{II}+2GI_{⊥}\right)}$$

where *I*_II_ and $I_{⊥}$ are fluorescence intensities observed in directions parallel and perpendicular, respectively, to the polarization direction of the exciting wave. *G* is an apparatus constant.

#### 3.4.2. Time-Resolved Fluorescence Spectroscopy

Emission intensities for time-resolved anisotropy of DPH probe (*I*_VV_(t) and *I*_VH_(t)) were obtained using reconvolution fitting of the measured decays (λ_EX_/λ_EM_ = 373/466 nm) with the following functions:

(3) $$\begin{array}{c} I_{\mathrm{VV}}\left(t\right)=G\overset{t}{\underset{-\infty }{\int }}\mathrm{IRF}\left(t^{\prime }\right)\frac{1}{3}\left(\alpha_{1}e^{-\frac{t-t^{\prime }}{\tau_{1}}}+\alpha_{2}e^{-\frac{t-t^{\prime }}{\tau_{2}}}\right)\left\{1+2\left[r_{\infty }+\left(r_{0}-r_{\infty }\right)e^{-\frac{t-t^{\prime }}{\varphi }}\right]\right\}dt^{\prime }\\ I_{\mathrm{VH}}\left(t\right)=\int_{-\infty }^{t}\mathrm{IRF}\left(t^{\prime }\right)\frac{1}{3}\left(\alpha_{1}e^{-\frac{t-t^{\prime }}{\tau_{1}}}+\alpha_{2}e^{-\frac{t-t^{\prime }}{\tau_{2}}}\right)\left\{1-\left[r_{\infty }+\left(r_{0}-r_{\infty }\right)e^{-\frac{t-t^{\prime }}{\varphi }}\right]\right\}dt^{\prime }, \end{array}$$

where IRF is the instrument response function measured for the scattering solution, $\alpha_{i}$ stands for the amplitude of the *i*^th^ fluorescence decay component with lifetime $\tau_{i}$, $r_{0}$ is the initial anisotropy, $r_{\infty }$ is the residual anisotropy, and φ is the lifetime (rotational correlation time) of the anisotropy decay. G-factor was determined by applying a tail matching method to the measured standard (ethanol solution of POPOP). The global fitting of the anisotropy decay parameters and the G-factor determination were performed using FluoFit v.4.5 (Pico Quant, Berlin, Germany).

The anisotropy data were evaluated using a model introduced by Kinosita, Kawato and Ikegami [42] in which DPH molecule wobbles uniformly in a lipid bilayer within a cone of semiangle $\theta_{c}$. the DPH orientational order parameter (*S*) was defined [43] as:

(4) $$S=\sqrt{\frac{r_{\infty }}{r_{0}}}=\frac{1}{2}\cos\theta_{c}\left(\cos\theta_{c}+1\right).$$

The so-called wobbling diffusion coefficient *D_w_*, defined by Kawato et al. [44], was calculated using the method of Lipari and Szabo [45]:

(5) $$\begin{array}{c} D_{W}=\frac{1}{\varphi }\cdot \frac{12x^{2}{\left(x+1\right)}^{2}\log\left(\frac{x+1}{2}\right)+7x^{6}-8x^{5}-22x^{4}+8x^{3}+17x^{2}+4x-6}{-6\left(x^{5}+x^{4}-x^{3}-x^{2}-4x+4\right)}\\ \mathrm{with}\;x=\cos\theta_{c}=\frac{\sqrt{8S+1}-1}{2} \end{array}$$

#### 3.4.3. Dynamic Light Scattering

The mean diameter and particle size distribution of three different types of LUVs composed of POPC, POPC with 5 mol % DOTAP and POPC with 5 mol % POPG were monitored as a function of time or concentration using dynamic light scattering (Zetasizer Nano ZS, Malvern Panalytical Ltd., Great Malvern, UK). The measurements were performed 15 min after addition of the compounds to the solution of liposomes and, if there was no aggregation, they were repeated every hour for next 8 h at 23 °C. The liposomes were suspended in phosphate buffer (pH 7.4, c = 0.5 mM) and modified by ethanolic solution of C, CG, GGA, GDG, CR, and CA used at 50 µM concentration. The added volume of ethanol did not exceed 5% *v*/*v* and did not affect measured sizes. The autocorrelation function of the scattered light was analyzed by the Cumulant method [46] to obtain the mean size (Z-AVG) and polydispersity index (PDI).

#### 3.4.4. Electrophoretic Light Scattering

Three different types of LUVs composed of the neutral lipids POPC, POPC with 5 mol% of the positively charged lipid DOTAP, and POPC with 5 mol % of the negatively charged POPG were used in these experiments. Test compounds were added to the dispersion of liposomes and the samples were incubated in the dark for 15 min at room temperature. By using electrophoretic light scattering (Zetasizer Nano ZS, Malvern Instruments, Malvern, UK), the electrophoretic mobility distribution of the liposomes was measured at 23 °C. On the basis of the frequency spectrum, obtained from digital correlation analysis, the electrical potential at the plane of shear, i.e., zeta potential (*ξ*), was calculated directly from the measured mobility by use of the Henry equation [47]:

(6) $$\zeta =\frac{3u\eta }{2\varepsilon_{0}\varepsilon f\left(\kappa_{a}\right)}$$

where: *µ* is the viscosity of the medium (0.8872), *u* is the measured electrophoretic mobility, $\varepsilon_{0}$ and ε are permittivities of the medium, and $f\left(\kappa_{a}\right)$ being 1.5 is the value of Henry function taken from the Smoluchowski equation [48].

#### 3.4.5. Fluorimetric Studies of the Antioxidant Activity of the Compounds

The antioxidant activity of the compounds towards POPC containing membranes was determined by fluorimetric methods using AAPH and H_2_O_2_ as oxidation inducers. Lipid peroxidation was detected with two probes: BODIPY^TM^ 655/676 and TMA-DPH. BODIPY is lipophilic probe that exhibits a change in fluorescence after interaction with peroxyl radicals generated by H_2_O_2_. The chromophore of TMA-DPH is located at the level of 4 carbon of lipids chain and its fluorescence exponentially decreases with increasing concentration of free radicals induced by homolytic decomposition of AAPH at 37 °C. Liposomes unmodified (control) and compounds-modified were suspended in a phosphate buffer of pH 7.4 and treated with the chemical oxidation inducer AAPH (20 mM) for 30 min or H_2_O_2_ (20 mM) for 3 h. Free radicals, released in the process of membrane lipid oxidation, cause quenching of the dyes, decreasing the fluorescence intensity. As a measure of the extent of lipid oxidation we used relative fluorescence, i.e., the ratio of florescence of the AAPH or H_2_O_2_-oxidized probe to the initial fluorescence of the probe. Here, as a control we used the relative fluorescence of POPC suspension that contained the dye, oxidized with AAPH or H_2_O_2_, while the blank was the relative fluorescence of a suspension of the same concentration but not oxidized. A Cary Eclipse (Varian Inc., Palo Alto, CA, USA) spectrofluorimeter was used to measure free radical concentrations in the samples. Excitation and emission wavelengths λ_EX_/λ_EM_ were 364/430 nm, and 655/676 nm for TMA-DPH and BODYPI, respectively. The measure of lipid oxidation was the relative change of the fluorescence intensity, *F*/*F*_0_, where *F*_0_ is the initial fluorescence and F the one measured during oxidation procedure. The percentage of lipid oxidation inhibition was calculated from the formula:

(7) $$\%\;of\;oxidation\;inhibition=\frac{\left(F_{x}-F_{u}\right)}{\left(F_{k}-F_{u}\right)}\cdot 100\%$$

where: *F_x_* = relative fluorescence of the sample oxidized by AAPH (or H_2_O_2_), for 30 min (or 3 h) in the presence of the compounds; *F_u_* = relative fluorescence of the control sample, oxidized by AAPH (or H_2_O_2_), without the compounds, measured after 30 min (or 3 h); *F_k_* = relative fluorescence of the blank sample, not subjected to oxidation procedure, measured after 30 min (or 3 h).

## 4. Conclusions

In this work, for the first time, the interaction of cyanidin and its O-glycosides with model lipid membrane composed of POPC was examined in detail. Obtained results allowed the determination and a better understanding of the mechanism of interaction of water-soluble vacuolar pigments with lipid membrane. Furthermore, on the basis of the antioxidant activity of the compounds and their influence on physical properties of lipid membranes a structure-activity relationship was determined. It was shown that the interaction of cyanidin with lipid membrane completely change after its O-glycosylation. The presence of sugar substituent(s) can change cyanidin antioxidant activity, inhibit its ability to aggregate lipid vesicles and significantly limit the depth of its penetration into the lipid bilayer. It was also shown that the ability of glycosides to induce changes in physical properties of the membrane depends on the type and the number of sugar substituents in the fallowing way: 3-O-glycosides containing monosaccharide induce greater changes than those containing disaccharides or more than one sugar substituent. The high antioxidant activity of the compounds and their influence on lipid membrane properties demonstrated in this work allows for their rational use in food industry and preventive healthcare. They will help in the design of functional foods, medicines and cosmetics enriched with water-soluble plant pigments of high biological activity.

## Figures and Tables

**Table 1:** Names, trade names, chemical structures and molecular characteristics of cyanidin and its O-glycosides tested in our study.

Abbr.	Name	Trade Name	Substitution Pattern		Structure *
			R_1_	R_2_	
**C**	Cyanidin	-	H	H	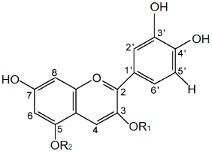
**CG**	Cyanidin-3-O-glucoside	Chrysanthemin	glucose	H	
**CR**	Cyanidin-3-O-rutinoside	Keracyanin	rutinose	H	
**CGA**	Cyanidin-3-O-galactoside	Ideain	galactose	H	
**CDG**	Cyanidin-3-5-O-di-glucoside	Cyanin	glucose	glucose	
**CA**	Cyanidin-3-O-arabinoside	-	arabinose	H	

* Flavynium cation.

## Supplementary Materials

The following are available online, Figure S1: The quenching of the fluorescence intensity of Laurdan probe caused by cyanidin and its O-glycosides used at concentration range from 25 µM to 100 µM. *I*_0_ and *I* are the fluorescence intensities of Lurdan probe measured at 440 nm before and after the addition of the tested compounds at room temperature, respectively. C—Cyanidin, CG—cyanidin-3-O-glucoside, CGA—cyanidin-3-O-galactoside, CA—cyanidin-3-O-arabinoside, CR—cyanidin-3-O-rutinoside, and CDG—cyanidin-3-5-O-diglucoside C, Figure S2: The relationship between anisotropy of DPH probe and the total number of OH groups in the structure of the compounds. The anisotropy was measured in 1-palmitoyl-2-oleoylphosphatidylcholine LUVs treated by: (A) cyanidin and its O-glycosides used at 50 µM concentration, at 35 °C; (B) cyanidin and its glycosides used at 100 µM concentration at 15 °C. The results of Pearson correlation analysis are added to the graphs: r-correlation coefficient, Figure S3: The values of Zeta potential of 1-palmitoyl-2-oleoylphosphatidylcholine LUVs treated by 100 µM of cyanidin (C) and its O-glycosides (CG—cyanidin-3-O-glucoside, CGA—cyanidin-3-O-galactoside, CA—cyanidin-3-O-arabinoside, CR—cyanidin-3-O-rutinoside, and CDG-cyanidin-3-5-O-diglucoside). The Zeta potential was measured 15 min after addition of the compounds, at 25 °C. Statistical significant differences between control and compounds modified LUVs are denoted: ** α = 0.01, Table S1: The Z-average diameters and polydispersity index values of LUVs liposomes measured 2 h after their modification by cyanidin used at different concentrations. The experiment was repeated thrice and the results are shown as mean value ± standard deviation.
