# Supplementary material for: The Impact of O-Glycosylation on Cyanidin Interaction with POPC Membranes: Structure-Activity Relationship
Source: Molecules. 2018 Oct 25;23(11):2771. doi: 10.3390/molecules23112771 (PMC6278410; doi:10.3390/molecules23112771)
Supplement: Supplementary file 1 [file molecules-23-02771-s001.pdf]

The impact of O-glycosylation on cyanidin interaction with POPC membrane. Structure-activity relationship

Sylwia Cyboran-Mikołajczyk[1], Piotr Jurkiewicz[2], Martin Hof[2], Halina Kleszczyńska[1]

[1]*Department of Physics and Biophysics, Wroclaw University of Environmental and Life Sciences, Norwida 25, 50-375 Wrocław, Poland, sylwia.cyboran@upwr.edu.pl, halina.kleszczyńska@upwr.edu.pl*

[2]*J Heyrovsky Institute of Physical Chemistry, Academy of Science of the Czech Republic, Dolejškova 2155/3, Prague 8, 182 23, Czech Republic, hof@jh-inst.cas.cz, piotr.jurkiewicz@jh-inst.cas.cz,*

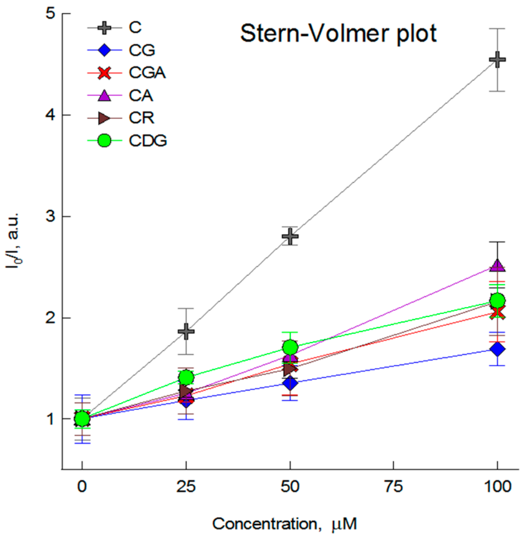

Figure 1S. The quenching of the fluorescence intensity of Laurdan probe caused by cyanidin and its O-glycosides used at concentration range from 25 μM to 100 μM. I_0 and I are the fluorescence intensities of Lurdan probe measured at 440 nm before and after the addition of the tested compounds at room temperature, respectively. C - Cyanidin, CG - cyanidin-3-O-glucoside, CGA - cyanidin-3-O-galactoside, CA - cyanidin-3-O-arabinoside, CR - cyanidin-3-O-rutinoside, and CDG - cyanidin-3-5-O-diglucoside C.

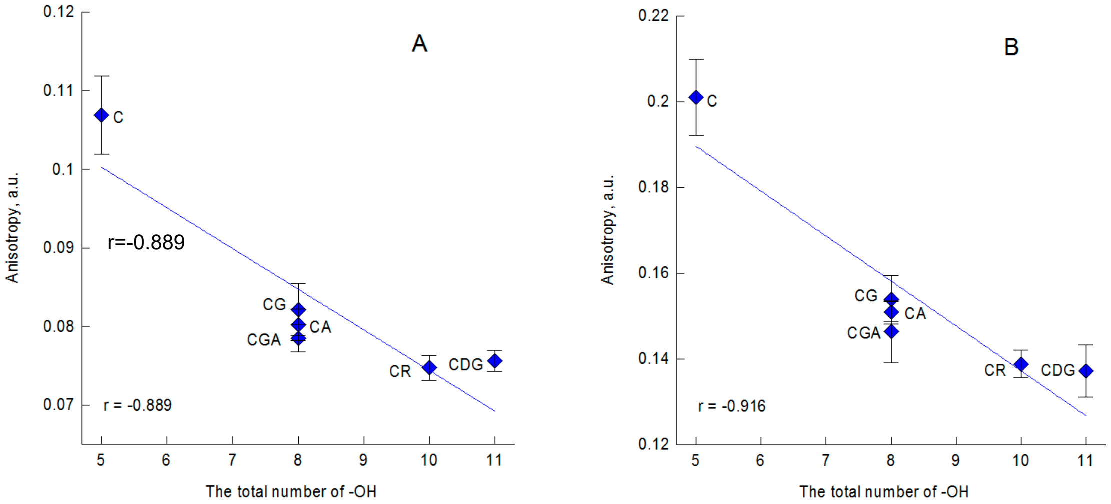

Figure 2S. The relationship between anisotropy of DPH probe and the total number of OH groups in the structure of the compounds. The anisotropy was measured in 1-palmitoyl-2-oleoylphosphatidylcholine LUVs treated by: A) cyanidin and its O-glycosides used at 50 μM concentration, at 35 °C; B) cyanidin and its glycosides used at 100 μM concentration at 15 °C. The results of Pearson correlation analysis are added to the graphs: r- correlation coefficient.

Table 1S. The Z-average diameters and polydispersity index values of LUVs liposomes measured 2 hours after their modification by cyanidin used at different concentrations. The experiment was repeated thrice and the results are shown as mean value ± standard deviation.

Concentration [μM]	Z-Average, nm	PDI, a.u.
0	137 ± 7.20	0.076 ± 0.004
0.1	137 ± 8.40	0.075 ± 0.005
0.5	139 ± 11.2	0.077 ± 0.007
1.0	152 ± 8.70*	0.121 ± 0.011*
2.0	342 ± 35.0**	0.321 ± 0.019**
2.5	514 ± 40.0**	0.525 ± 0.057**

The statistically significant differences between control and C-modified samples are denoted: $^{*}\alpha = 0.1$ and $^{**}\alpha = 0.05$.

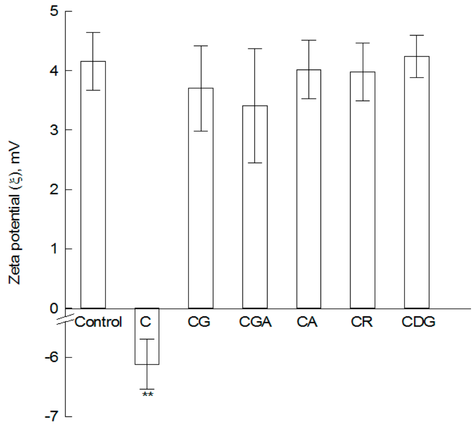

Figure 3S. The values of Zeta potential of 1-palmitoyl-2-oleoylphosphatidylcholine LUVs treated by 100 μM of cyanidin (C) and its O-glycosides (CG - cyanidin-3-O-glucoside, CGA - cyanidin-3-O-galactoside, CA - cyanidin-3-O-arabinoside, CR - cyanidin-3-O-rutinoside, and CDG - cyanidin-3-5-O-diglucoside). The Zeta potential was measured 15 min after addition of the compounds, at 25 °C. Statistical significant differences between control and compounds modified LUVs are denoted: ** α=0.01.

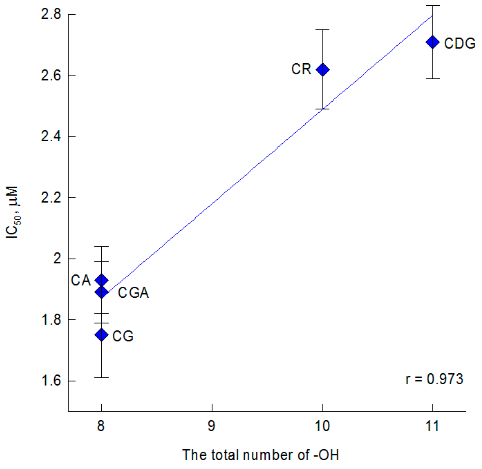

Figure 4S. The relationship between antioxidant activity of cyanidin glycosides (IC_{50}) and the total number of OH groups in the structure of the compounds. The POPC LUVs were oxidized by AAPH for 30 min. The IC_{50} is the concentration of the compound responsible for 50 % inhibition of lipid peroxidation. The results of Pearson correlation analysis are added to the graphs: r- correlation coefficient.
